# Evaporative Gasoline Emissions and Asthma Symptoms

**DOI:** 10.3390/ijerph7083051

**Published:** 2010-08-04

**Authors:** Mary Ellen Gordian, Alistair W Stewart, Stephen S Morris

**Affiliations:** 1 Institute of Social and Economic Research, University of Alaska Anchorage, 3211 Providence Dr., Diplomacy 504, Anchorage, AK 99508, USA; 2 Section of Epidemiology & Biostatistics, School of Population Health (Tamaki Campus), University of Auckland, Private Bag 92019, Auckland 1142, New Zealand; E-Mail: aw.stewart@auckland.ac.nz; 3 Air Quality Section, Department of Health and Human Services, Municipality of Anchorage, 632 West 6th Avenue, Anchorage, AK 99501, USA; E-Mail: morrisSS@ci.anchorage.ak.us

**Keywords:** MRLs, gasoline exposure, benzene, VOCs, aromatics

## Abstract

Attached garages are known to be associated with indoor air volatile organic compounds (VOCs). This study looked at indoor exposure to VOCs presumably from evaporative emissions of gasoline. Alaskan gasoline contains 5% benzene making benzene a marker for gasoline exposure. A survey of randomly chosen houses with attached garages was done in Anchorage Alaska to determine the exposure and assess respiratory health. Householders were asked to complete a health survey for each person and a household survey. They monitored indoor air in their primary living space for benzene, toluene, ethylbenzene and xylenes for one week using passive organic vapor monitoring badges. Benzene levels in homes ranged from undetectable to 58 parts per billion. The median benzene level in 509 homes tested was 2.96 ppb. Elevated benzene levels in the home were strongly associated with small engines and gasoline stored in the garage. High concentrations of benzene in gasoline increase indoor air levels of benzene in residences with attached garages exposing people to benzene at levels above ATSDR’s minimal risk level. Residents reported more severe symptoms of asthma in the homes with high gasoline exposure (16%) where benzene levels exceeded the 9 ppb.

## 1. Introduction

Over the past two decades volatile organic compounds (VOCs) measured in Alaska have had higher concentrations in both indoor and ambient air than most other cities in the United States [1]. Previous studies in Alaska have shown that attached garages are a significant source of benzene and other VOCs in the living space of the home. This project measured indoor VOCs, including benzene, in a random sample of homes with attached garages and investigated whether respiratory symptoms were associated with VOC exposure where the exposure was from evaporative emissions rather than combustion of gasoline.

Alaska is not required to use reformulated gasoline. The benzene content of Alaska gasoline is approximately 5% by volume, which is three to ten times higher than most gasoline used in the U.S. Material Safety Data Sheets from Alaskan refineries indicate that Alaska gasoline has a high content of all aromatics and has high volatility. Because benzene is a known carcinogen, it has been eliminated from household products, where toluene and xylenes are still in use. Benzene is a marker for gasoline exposure.

An indoor air study of 137 Anchorage homes performed in 1995 associated elevated benzene levels with the presence of attached garages, as well as parked motor vehicles, gasoline powered equipment and storage and use of fuel in the attached garages. Concentrations were highest in colder months when homes were tightly closed [2]. A study conducted by the University of Washington in 2004 showed that benzene, toluene, ethyl benzene, and xylenes (BTEX) concentrations in attached garages in Anchorage were typically 10 to 15 times higher than ambient levels. Tracer gas measurements from 45 homes showed that much of this highly polluted garage air was infiltrating into the living space of the house and was the most significant source of benzene in the home. On average, 27% of the air found inside the home was shown to have originated from the garage and attached garages accounted for more than 90% of the benzene found in the living space [3]. The contribution from garage air infiltration appears to be higher in Anchorage than other locations where similar measurements have been conducted. In 15 houses with attached garages in southeast Michigan, infiltration from the garage accounted for 6.5 ± 5.3% of total air exchange and 50–60% of total BTEX in the living space [4]. Among 11 houses examined in a Boston area study, at the median, approximately 40% of the BTEX was attributable to sources in the attached garage. [5].

It is clear that VOCs can significantly affect respiratory health. Chamber studies show that healthy people when exposed to VOCs at less than 50 mg/cubic meter of air for four hours developed dose-related increases in lower respiratory and upper respiratory symptoms, with no significant change in lung function [6]. Children living in the Kanawha valley West Virginia, USA reported increased respiratory symptoms in relation to measured VOC exposure [7]. A recent study using National Health and Nutrition Examination Survey (NHANES) and personal VOC monitoring found that persons exposed to environmental aromatic VOCs had an odds ratio of 1.63 (1.17–2.27) of having doctor-diagnosed asthma [8].

Roadways are associated with increased PM-2.5 but they are also a common source of VOCs. Children living in close proximity to roadways have more respiratory symptoms [9–13], decreased lung function [13–15], more respiratory hospitalizations [16] and increased symptoms of asthma [17–21]. Children exposed to VOCs in their homes had increased risk of hospitalizations for asthma [22]. Recently two studies from very different locations have both shown that children without a family history of asthma are much more likely to develop asthma if they are living close to heavy traffic areas [23,24].

Environmental exposure to VOCs is a common occurrence, but difficult to study because of confounding by other pollutants and the difficulty in establishing exposure levels. This study was done to determine the extent of exposure to gasoline fumes that was occurring within homes with attached garages. The study also addressed whether there might be respiratory health effects from such exposure. It is expected that this approach would reduce confounding by fine particulates, road dust and the combustion products of fuel such as nitrogen and sulfur oxides.

## 2. Methods

After approval from the Institutional Review Board, tax records from the approximately 70,000 residential units in the Municipality of Anchorage were sorted to identify single family homes with attached garages that were occupied by the owner. There were approximately ten thousand such houses in the Municipality of Anchorage. In November 2008 a random sample of 1,700 homeowners was mailed a recruitment letter soliciting their participation in the study. A second recruitment letter was sent to those in the same group who had not responded.

A total of 571 (34%) households agreed to participate. Of these, 509 successfully completed monitoring within their home. Sampling was conducted during the winter season (November 2008 through April 2009) as the acceptances came in. Participating households were required to complete a short survey to document basic architectural and mechanical features of their home (square footage, heating system type, year built, *etc.*) and to confirm they had an attached garage. The survey asked whether the garage was used for parking cars and whether containers of gasoline and/or solvents were stored inside. Respondents were also asked to provide information on the number and type of gasoline-fueled equipment (e.g., lawnmowers, chainsaws, generators, snowmobiles, ATVs, snow blowers, *etc.*) in the garage.

Participating households were instructed to deploy a 3M™ OVM 3500 passive organic vapor monitoring badge in their primary living area for one week. Specific instructions were provided to each household about how to deploy the badge and properly document the sample. Every tenth household was sent two badges to deploy simultaneously to assess sampling precision. In preparation for this study, OVM 3500 passive badge measurements were compared with the EPA Method TO-15 Summa canister method. Collocated canisters and OVM 3500 badges were found to consistently agree within 15% for all BTEX compounds.

All organic vapor monitors were delivered to the Applied Science and Environmental Technology (ASET) laboratory at the University of Alaska Anchorage. The target compounds (benzene, toluene, ethyl benzene and the xylenes) were evaluated using the National Institute for Occupational Safety and Health protocol (EPA Compendium Method TO-17). The charcoal filters were extracted using 1 mL of carbon disulfide containing 5 ug/mL 4-bromofluorobenzene as the internal standard. The extraction solvent was freshly prepared for each set of samples analyzed. Each charcoal pad was placed inside a 2 mL amber vial, 1 mL of the extraction solvent was added and the vials closed with Teflon lined caps. The pads were desorbed for 40 min in an ultrasonic bath with water maintained at 15–18 °C. After sonication the extract was withdrawn and transferred to an amber autosampler vial. Working analytical standards in the range of 0.1–10 ug/mL were prepared using the solvent plus ISTD for dilution of commercial standard solutions. All extracts and standards were analyzed by GC/MS using a Varian 3800 Gas Chromatograph equipped with a Varian 2200 Ion Trap detector. The separation was accomplished using an RTX-1 60 m × 0.25 mm id column with a 1 mm film thickness.

Data quality was evaluated through the analysis of field and laboratory blanks, and by analyzing replicate samples collected from 10% of participating households.

The health survey related to respiratory symptoms included eight questions modeled on questions from the International Study of Asthma and Allergies in Childhood (ISAAC) although they were modified to be applicable to adults as well as children. Parents answered for their children.

*Has this person* ***EVER*** *had wheezing or whistling in his/her chest at any time?*
If yes, in the last 12 months, how many attacks of wheezing?
How often has this person awakened from sleep with wheezing?
Has wheezing ever been severe enough to limit his/her speech to 1 or 2 words between breaths?
Has his/her chest ever sounded wheezy during or after exercise?
Has this person used medications such as inhalers or pills to help breathing?
Has this person had a dry cough at night without a cold or chest infection?

Severe asthma was identified by having either four or more wheezing attacks per year, one or more nights per week sleep disturbance due to wheezing or difficulty speaking during an attack. Because all of the aromatic VOCs that were measured were highly correlated with each other we decided to use benzene as the exposure of interest because the Agency for Toxic Substances and Disease Registry (ATSDR) has established minimal risk levels (MRLs) for non-cancer health effects for inhalation exposure to benzene that were within the range of the exposures that we had found. The MRLs provide a basis for discussion of health effects of exposure. There is no minimal risk levels set for gasoline because gasoline composition varies over time and location. The MRLs for benzene were set only for non-cancer outcomes. We established three exposure groups based on MRLs as follows:

- High exposure >9 ppb of benzene
- Intermediate exposure ≥3 ppb and ≤9 ppb of benzene
- Low exposure <3 ppb of benzene

The “high exposure” group corresponds with ATSDR’s MRL designation for acute duration inhalation exposure (≤14 days). The “low exposure” group is below the ATSDR MRL for chronic exposure (>365 days) [25]. That is to say, that if the house remains below 3 ppb of benzene over the entire year the exposure is below the minimal risk level for benzene.

The modeling for the analyses was done by using a generalized linear mixed model (GLMM) to account for the correlation based on number of persons in each household. This was a logistic model with the households being modeled as a random effect. The odds of reporting possible asthma-related symptoms in the survey were tabulated for each group. Odds ratios were computed to contrast the likelihood of subjects reporting symptoms in the high and intermediate exposure relative to the low exposure group.

## 3. Results and Discussion

Of the 1,700 households invited, 571 (34%) households containing in total 1,584 individuals said they would participate. Six hundred and twenty responded. Households who declined to participate but returned the letter stated they would be on vacation, moving or were too busy to participate during the sampling period. All participants had lived in their homes more than one month. Five hundred nine households containing 1,484 persons completed the surveys and measured aromatics in their homes for at least one week.

Caucasians were the predominant (85.1%) ethnic group. Eighty-five persons (5.7%) identified themselves as Asian or Pacific Islander. All other races were less than two percent except mixed race category of 57 persons (3.8%). Table 1 gives a summary of the demographics of the sample.

The recruitment targeted only homeowners with attached garages hence the average annual income for the households ($100,000 plus) was higher than the average income for households in Anchorage. Most houses were heated with natural gas (94%) and had forced air heat (89%).

Field blanks and laboratory blanks were all below detection limits for BTEX. For benzene, the coefficient of variation between replicate sample pairs averaged 14.7%.

BTEX concentrations among the 509 homes were skewed to the left. The median concentration was 2.9 ppb. Forty seven percent (47%) of the homes had benzene levels greater than 3 ppb. Fifty-five homes or 10.8% had benzene levels below the limits of detection. Sixteen percent of the homes had levels greater than 9 ppb measured over one week which exceeds the ATSDR minimal risk level for acute exposure. Descriptive statistics for BTEX measurements are given in Table 2.

**Figure 1.**
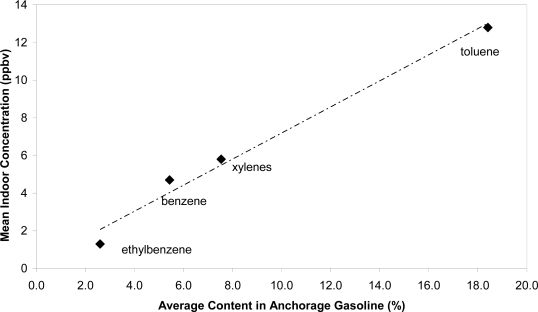
Comparison of Anchorage gasoline BTEX composition with concentration in indoor air.

Figure 1 shows that the relative concentration of each of the BTEX compounds found indoors closely mirrored the relative composition of the various BTEX compounds found in gasoline, providing strong evidence that gasoline was the primary source of these compounds in the study homes. The average content of each of the BTEX compounds in gasoline was determined from analyses of approximately 150 samples of regular and premium gasoline sampled from 20 Anchorage stations in October 2008–September 2009. Indoor concentrations of the various BTEX compounds were also highly correlated with each other (r^2^ > 0.9).

**Figure 2.**
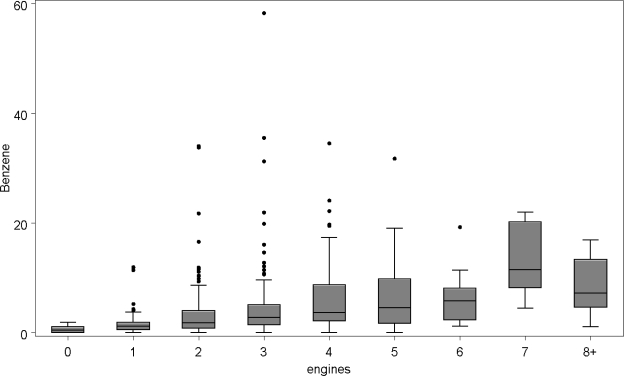
Benzene levels in ppb *vs.* total number of engines kept in the garage.

The relationship between the benzene concentration in each home and its architectural and mechanical features was examined; there was no statistically significant association between benzene and the size, nor age of the home, nor with the type of heating system used. The presence of smokers in the household was not related to the concentration of benzene. Benzene concentrations were, however, strongly positively associated with the presence of portable gasoline storage containers. The number of automobiles kept in the garage was suggestive of an association but not statistically significant, but the total number of engines (Figure 2) which included small engines plus vehicles that were kept in the garage was strongly correlated with the median level of benzene exposure. The line in the box of the boxplot indicates the median benzene level.

Households and individuals were divided by their benzene exposure over the one week of monitoring into low exposure (<3 ppb), intermediate exposure (3–9 ppb), and high exposure (>9 ppb). The numbers within these categories are in Table 3.

Eight health measures were modeled to assess whether there was a difference in the proportion of those reporting particular health outcomes in the homes with low benzene as compared with those who had intermediate and high benzene exposures in their homes. Possible confounders such as age, exposure to gasoline at work or during leisure activities (e.g., boating, snowmobiling, *etc.*) and the presence of pets, molds and/or smokers in the home were included in the model. The interrelationship of being in household clusters was accounted for in the model. Table 4 shows the percentage at each exposure level having the symptom and the odds ratio of the high and intermediate exposure groups relative to the low exposure group. Odds ratios were greater than one for all health measures except sleep disturbance, indicating that these symptoms were more prevalent in individuals living in houses with intermediate and high exposures to benzene.

There was however, a significant association between elevated benzene and those exhibiting severe symptoms related to wheezing. Individuals in the high exposure group were 2.5 times more likely to have severe asthma symptoms than those in the low exposure group.

## 4. Discussion

This study measured the indoor air benzene, toluene, ethylbenzene and xylenes (BTEX) in over 500 residences with attached garages in Anchorage, Alaska. The relative proportion of BTEX measured in the indoor air mirrored the relative composition of BTEX compounds in Alaska gasoline, supporting gasoline as the primary source of BTEX in homes.

Because benzene, a carcinogen, is not used in household products, it was used as a marker of gasoline exposure. Indoor air benzene was related strongly to the number of gasoline powered engines that were in the garage and with the presence of gasoline storage containers in the garage.

The study results show that 16% of the houses with attached garages studied in Anchorage had indoor air benzene that exceeded the ATSDR minimal risk level (MRL) for acute exposure to inhaled benzene. This indicates that a substantial proportion of people who live in homes with attached garages are being exposed to gasoline fumes and elevated indoor benzene levels when the gasoline contains benzene at levels of 5% by volume.

Not all attached garages are equal. Some garages have better vapor barriers or better ventilation than others and allow less gasoline fumes to enter the house. Storing gasoline and gasoline engines (lawn mowers, ATVs, snowmobiles, generators, *etc.*) in the garage are behaviors entirely dependent on the householder. Some people elect to store their gasoline and gasoline engines in storage sheds outside the garage-house airspace. Smoking contributed very little to the findings because only 46 (9%) of households included a smoker and only 55 (4%) of the participants identified themselves as smokers.

Although participants were randomly recruited from tax records, the participation rate was only 34% of the homes indicating that the households might not be representative of all Anchorage homes with attached garages. Participation bias could skew the prevalence of asthma among participants, but is unlikely to confound a potentially related association with BTEX exposure. The prevalence of asthma measured in this study was consistent with the rates of asthma diagnosis reported by the Behavioral Risk Factor Surveillance System (BRFSS) in Alaska in 2009.

The survey and BTEX measurements were completed by the homeowners with only written instructions from the researchers. It is possible that some homeowners placed the badges in areas of particularly high or low expected exposure not in the main living area at breathing level as they were instructed.

The ATSDR minimal risk levels are set for exposure over a period of time. We measured the VOCs in the indoor air for one week during the winter months. The result may not be representative of exposure over a longer period of time or a different season.

None of the survey answers were confirmed from medical records, nor were any physical health measurements done. Asthma diagnosis was a lifetime diagnosis and hence may not be related to the current residence. However, there was no increase in asthma diagnosis in children who were likely to have spent a larger proportion of their lifetime in the residence. Medication use could reduce the number of cases of severe asthma, however, participants were toward the upper end of the economic range and should have equal access to medication.

Measuring indoor air exposure in an environment where 94% of homes were heated with natural gas and the other 6% by electricity should reduce the confounding effects of fine particulates from combustion in this study. Benzene levels were a surrogate for gasoline exposure in this study, but it is unknown whether benzene, the mixture of aromatics, or other gasoline components are associated with the increased risk of severe asthma.

The odds ratios did not reach statistical significance for the effects measured except for severe asthma; however, all but one of the odds ratios were positive for an exposure effect which would be unlikely if there were no effect of exposure. This study did not have sufficient power to look at the effects on vulnerable populations such as infants or elderly.

## 5. Conclusions

High concentrations of benzene in gasoline increase the risk of high indoor air levels of benzene in residences likely exposing a large part of the population where this occurs to benzene at levels above the minimal risk. Increased severity of asthma occurred when benzene levels exceeded 9 ppb although this may be related to the mixture of VOCs found in gasoline and not to benzene itself.

## Figures and Tables

**Table 1.** Demographics of the participants.

**Demographic**	**Category**	**Number (%)**
Gender	Female Male Missing	761 (50.8%) 719 (48.0%) 4 (0.2%)
Race	Caucasian Asian/PI Mixed race Black Am.I/Alaska Native Other Missing	1,263 (85.1%) 82 (5.5%) 55 (3.7%) 26 (1.8%) 26 (1.8%) 24 (1.6%) 8 (0.5%)
Age	0–19 20–65 66+ missing	458 (30.6%) 1,010 (67.5%) 13 (0.9%) 3 (0.2%)
Employment	Yes No	826 (55.7%) 658 (44.3%)
Current Smokers (Individuals)	Yes No missing	55 (4%) 1,424 (96%) 5 (0.3%)
Smokers live in household (Households)	Yes No missing	46 (8.9%) 463 (89.9%) 6 (1.2%)

**Table 2.** Descriptive statistics for indoor air BTEX analysis.

		**Benzene**	**Toluene**	**Ethylbenzene**	**Xylenes**

N		509	509	509	509
Median (ppb)		2.88	7.34	0.83	3.01
Minimum (ppb)		<0.4	<1.8	<0.7	<2.5
Maximum (ppb)		58.29	179.17	13.74	77.26
Concentration by Percentiles (ppb)	25th	1.19	3.03	0.34	<2.5
	75th	5.92	14.68	1.74	8.05
	90th	11.16	27.36	3.15	14.14

**Table 3.** Benzene Exposure by Grouping.

Exposure	Individuals	Households*	Median benzene level
Low <3ppb	791	275	1.25 ppb
Intermediate 3–9 ppb	439	162	4.75 ppb
High > 9 ppb	252	81	12.27 ppb

* Nine households measured BTEX but did not complete health surveys.

**Table 4.** Odds ratios for reporting symptoms relative to low exposure < 3 ppb.

	**% reporting symptom within each exposure group**			**Odds ratio of having the symptom with the referent being low exposure <3 ppb**	
**Symptom**	**Low exposure <3 ppb N = 787**	**Intermediate exposure (3–9 ppb) N = 441**	**High exposure >9 ppb N = 252**	**Intermediate Exposure (3–9 ppb)** Odds ratio (95% CI)	**High Exposure (>9ppb)** Odds ratio (95% CI)
Wheeze	12.8	13.7	14.2	1.03 (0.63,1.68)	1.15 (0.63,2.09)
Asthma attacks	4.0	4.1	6.5	1.06 (0.52,2.17)	1.80 (0.80,4.06)
Wheezing Sleep disturbance	0.8	1.2	0.8	1.23 (0.32,4.64)	1.00 (0.17,5.88)
Exercise-induced asthma	11.8	13.7	15.4	1.28 (0.76,2.13)	1.48 (0.80,2.76)
Dry Cough	14.1	14.2	17.9	1.06 (0.64,1.74)	1.49 (0.81,2.73)
Diagnosed Asthma	12.4	13.1	12.5	1.04 (0.67,1.63)	1.06 (0.61,1.85)
Allergies	30.8	31.0	30.8	1.11 (0.77,1.61)	1.13 (0.72,1.79)
Severe asthma *	5.4	6.3	10.6	1.34 (0.70,2.54)	2.49 (1.22,5.07)

* Severe asthma was identified by having either four or more wheezing attacks per year, one or more nights per week sleep disturbance due to wheezing, or difficulty speaking during an attack.

## References

[1] Air Quality in Anchorage: A Summary of Air Monitoring Data and Trends (1980–2008). March 2009; Available online: http://www.muni.org/Departments/health/environment/AirQ/Documents/2009%20report%20final.pdf/ (accessed on 23 April 2010)

[2] Schlapia A Morris S Architectural, behavioral and environmental factors associated with VOCs in Anchorage homes Proceedings of the 91st Annual Meeting of the Air & Waste Management Association San Diego, CA, USA, date Month1998; Document # 98-A504; Available online: http://www.muni.org/Departments/health/environment/Adobe%20Documents%20for%20ESD%20Site/Architectural%20Behavioral%20and%20Environmental%20Factors%201996.pdf (accessed on 26 April 2010)

[3] Morris S Influence of Attached Garages on Indoor VOC Concentrations in Anchorage Homesproceeding of the Annual Meeting of Northwest International Section of the Air & Waste Management Association Seattle, WA, USA 2004 Available online: http://www.pnwis.org/PNWIS2004/Presentations/3.3.3%20Morris%20Indoor%20Air%20Q%20of%20Attached%20Garages.pdf (accessed on 23 April 2010)

[4] Batterman S Chunrong J Hatzivasilis G Migration of volatile organic compounds from attached garages to residences: A major exposure source Environ. Research 2007 104 224 240 10.1016/j.envres.2007.01.008 17350611

[5] Dodson RE Levy JI Spengler JD Shine JP Bennett DH Influence of basements, garages and common hallways on indoor residential volatile organic compound concentrations Atmos. Environ 2008 42 1569 1581

[6] Pappas GP Herbert RJ Henderson W Koenig JQ Stover B Barnhart S The respiratory effects of volatile organic compounds Int. J. Occup. Environ. Health 2000 6 1 8 10637531 10.1179/oeh.2000.6.1.1

[7] Ware JH Spengler JD Neas LM Samet JM Wagner GR Coultas D Ozkaynak H Schwab M Respiratory and irritant health effects of ambient volatile organic compounds Am. J. Epidemiol 1993 137 1287 1301 8333411 10.1093/oxfordjournals.aje.a116639

[8] Arif AA Shah SM Association between personal exposure to volatile organic compounds and asthma among US adult population Int. Arch. Occup. Environ. Health 2007 80 711 719 17357796 10.1007/s00420-007-0183-2

[9] Brauer M Hoek G van Vliet P Meliefste K Fischer PH Wijga A Koopman LP Neijens HJ Gerritsen J Kerkhof M Heinrich J Bellander T Brunekreef B Air pollution from traffic and the development of respiratory infections and asthmatic and allergic symptoms in children Am. J. Resp. Crit. Care Med 2002 166 1092 1098 12379553 10.1164/rccm.200108-007OC

[10] Ciccone G Forastiere F Agabiti N Biggeri A Bisanti L Chellini E Corbo G Dell’Orco V Dalmasso P Volante TF Galassi C Piffer S Renzoni E Rusconi F Sestini P Viegi G Road traffic and adverse respiratory effects in children. SIDRIA Collaborative Group Occup. Environ. Med 1998 11 771 778 9924455 10.1136/oem.55.11.771 PMC1757532

[11] Kim JJ Smorodinsky S Lipsett M Singer BC Hodgson AT Ostro B Traffic-related air pollution near busy roads: the East Bay Children’s Respiratory Health Study Am. J. Resp. Crit. Care Med 2004 170 520 526 15184208 10.1164/rccm.200403-281OC

[12] Shima M Nitta Y Adachi M Traffic-related air pollution and respiratory symptoms in children living along trunk roads in Chiba Prefecture, Japan J. Epidemiol 2003 3 108 119 12675120 10.2188/jea.13.108 PMC9588432

[13] Thompson AJ Shields MD Patterson CC Acute asthma exacerbations and air pollutants in children living in Belfast, Northern Ireland Arch. Environ. Health 2001 56 234 241 11480499 10.1080/00039890109604447

[14] Brunekreef B Janssen NA de Hartog J Harssema H Knape M van Vliet P Air pollution from truck traffic and lung function in children living near motorways Epidemiology 1997 8 298 303 9115026 10.1097/00001648-199705000-00012

[15] Delfino R Gong H Linn WS Hu Y Pellizzari ED Respiratory symptoms and peak expiratory flow in children with asthma in relation to volatile organic compounds in exhaled breath and ambient air J. Expos. Anal. Environ. Epidemiol 2003 13 348 363 10.1038/sj.jea.7500287 12973363

[16] Edwards J Walters S Griffiths RK Hospital admissions for asthma in preschool children: relationship to major roads in Birmingham, United Kingdom Arch. Environ. Health 1994 49 223 227 7518223 10.1080/00039896.1994.9937471

[17] Brunekreef B Stewart AW Anderson HR Lai CKW Strachan DP Pearce N ISAAC Phase 3 Study Group Self-Reported Truck Traffic on the Street of Residence and Symptoms of Asthma and Allergic Disease: A Global Relationship in ISAAC Phase 3 Environ. Health Perspect 2009 117 1791 1798 20049134 10.1289/ehp.0800467 PMC2801184

[18] Duhme H Weiland SK Keil U Kraemer B Schmid M Stender M Chambless L The association between self-reported symptoms of asthma and allergic rhinitis and self-reported traffic density on street of residence in adolescents Epidemiology 1996 7 578 582 8899382 10.1097/00001648-199611000-00003

[19] van Vliet P Knape M de Hartog J Janssen N Harssema H Brunekreef B Motor vehicle exhaust and chronic respiratory symptoms in children living near freeways Environ. Res 1997 74 122 132 9339225 10.1006/enrs.1997.3757

[20] Venn AJ Lewis SA Cooper M Hubbard R Britton J Living near a main road and the risk of wheezing illness in children Am. J. Resp. Crit. Care Med 2001 164 2177 2180 11751183 10.1164/ajrccm.164.12.2106126

[21] Zmirou D Gauvin S Pin I Momas I Sahraoui F Just J Le Moullec Y Brémont F Cassadou S Reungoat P Albertini M Lauvergne N Chiron M Labbé A Vesta investigators. Traffic related air pollution and the incidence of childhood asthma: results of the Vesta case-control study J. Epidemiol. Community Health 2004 58 18 23 14684722 10.1136/jech.58.1.18 PMC1757023

[22] Rumchev K Spickett J Bulsara M Phillips M Stick S Association of domestic exposure to volatile organic compounds with asthma in young children Thorax 2004 59 746 751 15333849 10.1136/thx.2003.013680 PMC1747137

[23] Gordian ME Haneuse S Wakefield J An investigation of the association between traffic exposure and the diagnosis of asthma in children J. Expo. Sci. Environ. Epidemiol 2006 16 49 55 16007113 10.1038/sj.jea.7500436

[24] McConnell R Berhane K Yao L Jerrett M Lurmann F Gilliland F Künzli N Gauderman J Avol E Thomas D Peters J Traffic, susceptibility, and childhood asthma Environ. Health Perspect 2006 114 766 772 16675435 10.1289/ehp.8594 PMC1459934

[25] Agency for Toxic Substances and Disease Registry Minimal Risk Levels. 1 Sept 2009; Available online: http://www.atsdr.cdc.gov/mrls/mrls_list.html/ (Accessed on 23 April 2010)

